# A rare case of antidepressant discontinuation syndrome triggered by domperidone: Clinical insights and literature review

**DOI:** 10.1177/2050313X251342973

**Published:** 2025-05-24

**Authors:** Ashour Barkho, Bushra Elhusein, Niman Gajebasia

**Affiliations:** 1 Department of Pharmacy, London Health Sciences Centre, ON, Canada; 2 Department of Psychiatry, London Health Sciences Centre, ON, Canada; 3 Assistant Professor of Psychiatry, Schulich School of Medicine and Dentistry, University of Western Ontario, London, ON, Canada

**Keywords:** antidepressant discontinuation syndrome, brain zaps, desvenlafaxine, domperidone, SNRIs

## Abstract

Antidepressant discontinuation syndrome is commonly observed among patients who abruptly discontinue or reduce the dosage of an antidepressant that has been administered for at least 6 weeks. Common manifestations may include flu-like symptoms, insomnia, nausea, impaired balance, sensory disturbances, headache, irritability, anxiety, and hyperarousal. These symptoms are typically mild, persist for 1–2 weeks, and resolve upon resumption of the medication. The probability of developing this syndrome increases with the prescribed pharmacological agent’s prolonged treatment duration and shorter half-life. Before prescribing antidepressants, it is imperative to inform patients about the potential complications associated with the sudden discontinuation of the medication. Understanding this phenomenon can help prevent future incidents and reduce nonadherence risk. The majority of antidepressants possess the potential to induce antidepressant discontinuation syndrome. This case report presents a unique instance of discontinuation syndrome in a 50-year-old female patient with major depressive disorder who had been effectively managed on desvenlafaxine for several years. After initiating domperidone for chemotherapy-induced nausea, she experienced “brain zaps” and other symptoms consistent with antidepressant discontinuation syndrome. Upon cessation of domperidone, her symptoms rapidly resolved. This report examines potential interactions between domperidone and desvenlafaxine, emphasizing the necessity for clinicians to be cognizant of possible drug interactions that may precipitate discontinuation symptoms.

## Introduction

Antidepressant discontinuation syndrome (ADS) is a group of symptoms that manifest following the abrupt cessation or dose reduction of antidepressant medications, particularly those affecting serotonin and norepinephrine reuptake, such as selective serotonin reuptake inhibitors (SSRIs) and serotonin–norepinephrine reuptake inhibitors (SNRIs).^1,2^

ADS has been increasingly scrutinized in recent years, particularly concerning its incidence rates among individuals ceasing antidepressant treatment. Recent studies suggest a notable variability in the prevalence of withdrawal symptoms, with some meta-analyses indicating that ~41%–56% of patients discontinuing SSRIs experience significant withdrawal symptoms.^3,4^ In a systematic review, mainly focusing on patients withdrawing from various classes of antidepressants, the incidence rates were found to hover around 30%–40% for those on different antidepressants, including SNRIs.^5^ Furthermore, a cohort study reported that among long-term users of SSRIs, nearly 50% experienced adverse effects following cessation, highlighting the clinical relevance of this syndrome.^6^ The considerable variation in these estimates may be attributed to differences in study design, patient populations, types of antidepressants, and definitions of discontinuation syndrome. Some other factors influencing these rates include the duration of antidepressant use, dosage, and the presence of preexisting psychiatric conditions, suggesting that the likelihood of encountering ADS may be modulated by individual patient characteristics and the specific medication regimen being utilized.^7^

Common ADS symptoms include dizziness, headaches, nausea, irritability, insomnia, sensory disturbances (e.g. brain zaps), and anxiety.^8^ These symptoms are thought to result from rapid changes in neurotransmitter levels, particularly serotonin, following the withdrawal of antidepressant medications.^1^ SNRIs increase serotonin and norepinephrine levels in the brain by inhibiting their reuptake into nerve cells. Upon discontinuation or significant dose reduction of an SNRI, the decrease in serotonin and norepinephrine levels can induce a transient imbalance in brain chemistry. The neural networks that depend on these neurotransmitters may experience difficulty adapting to these alterations, potentially resulting in anomalous sensations such as brain zaps.^9^

An alternative hypothesis regarding the etiology of brain zaps posits a mechanism involving serotonin receptor sensitivity. Discontinuation of desvenlafaxine, an SNRI, may restore serotonin reuptake activity, which, in the context of desensitized postsynaptic 5-HT1A receptors, could contribute to an acute hypo-serotonergic state, potentially manifesting as discontinuation symptoms.^9^ This excessive stimulation or abrupt cessation of normal serotonin signalling may precipitate neurological phenomena such as brain zaps.

Desvenlafaxine is an antidepressant that belongs to a class of drugs known as SNRIs. Desvenlafaxine is the principal active metabolite of venlafaxine. Consequently, the two agents exhibit structural similarity and are characterized by two non-adjacent chemical rings. In vitro studies have demonstrated that desvenlafaxine exhibits a 10-fold greater serotonin selectivity than norepinephrine.^10^ Desvenlafaxine is commonly prescribed for major depressive disorder, among other conditions, supported by its established efficacy.^11^ It is known to have a relatively favourable side-effect profile compared to older antidepressants, such as tricyclics, due to reduced anticholinergic and cardiotoxic effects.^12^ However, similar to other SNRIs, desvenlafaxine is associated with an increased risk of ADS upon abrupt discontinuation or dose reduction.^13^ The syndrome is typically managed by gradually tapering off the antidepressant or reintroducing the medication when symptoms become severe.^2^ In clinically comparable scenarios, a gradual dose reduction or a more progressive taper can effectively minimize the sudden drop in desvenlafaxine levels, thereby reducing the risk and severity of discontinuation symptoms.^14^

Desvenlafaxine is well known for its short half-life, a factor influencing the onset and severity of withdrawal symptoms. Gastaldon et al. identified that antidepressants with shorter half-lives are more frequently associated with reports of withdrawal syndrome, thereby highlighting the relationship between desvenlafaxine’s pharmacokinetic profile and the propensity for discontinuation syndrome.^15^ Furthermore, a systematic review by Fava et al. indicates that the cessation of venlafaxine and its derivative, desvenlafaxine, often results in significant neuropsychological and somatic symptoms, even when tapering is conducted gradually.^16^

On the other hand, domperidone is a peripheral dopamine D2 receptor antagonist commonly used to manage nausea, vomiting, and gastrointestinal motility disorders.^17^ It does not cross the blood–brain barrier significantly and is generally considered to have minimal central nervous system (CNS) effects.^17^ However, dopamine receptors play a modulatory role in regulating serotonin and norepinephrine systems, and interactions between dopaminergic and serotonergic pathways are well documented.^18^ For example, dopamine receptor antagonists have been shown to influence serotonin release in certain brain regions, potentially affecting mood and anxiety.^18,19^ Furthermore, dopamine antagonists primarily exert their effects by blocking dopamine D2 receptors, which, in the case of domperidone, occurs predominantly in the periphery due to its limited ability to cross the blood–brain barrier. Dopamine and serotonin systems are known to interact in the CNS, where dopamine antagonism can modulate serotonin release indirectly, for example, through feedback loops involving the raphe nuclei or prefrontal cortex.^20^ However, these interactions are typically studied with centrally acting dopamine antagonists (e.g. antipsychotics), not peripherally selective agents such as domperidone.^21^ Similarly, norepinephrine release might be affected indirectly via dopaminergic modulation of locus coeruleus activity, but evidence for this with domperidone is lacking due to its minimal central penetration.

In our specific example, there is no research data that has investigated or examined the drug–drug interaction between desvenlafaxine and domperidone, making this case report unique in addressing this issue and opening the door for future studies to shed further light on this in the future. While dopamine receptor antagonists may theoretically influence serotonin and norepinephrine dynamics, the relationship between peripherally acting agents like domperidone and SNRIs such as desvenlafaxine is not well established. We speculate that interaction, if present, could involve subtle peripheral effects on drug bioavailability or uncharacterized indirect effects, though this requires further investigation.

This case report describes a patient on stable desvenlafaxine therapy who developed discontinuation syndrome following the initiation of domperidone. The temporal relationship between domperidone use and symptom onset, as well as symptom resolution upon discontinuation of domperidone, suggests a potential interaction between these medications. This case underscores the need for clinicians to be aware of possible drug interactions involving dopamine antagonists and SNRIs, even in the absence of direct pharmacological overlap. A literature review is provided to explore potential mechanisms and contextualize this finding.

## Case presentation

A 50-year-old female patient was referred to mental health services for diagnostic clarification and medication adjustments after two decades of treatment for depression and anxiety with various antidepressants. Before her recent engagement with specialized psychiatric services, she had tried different medications without apparent results. She had been on venlafaxine at a maximum dosage of 225 mg for several years. Still, she recently reported that her depressive symptoms were not well-controlled, prompting her referral for further evaluation. While attempting to taper off venlafaxine, she experienced “brain zaps” during the reduction process.

During the initial psychiatric consultation, the patient reported persisting depressive symptoms, leading to a decision to switch from venlafaxine to a different antidepressant. After multiple trials, her condition responded positively to desvenlafaxine, which has since been gradually increased to a daily dosage of 200 mg. The patient has maintained stability and improved depressive symptoms, managing desvenlafaxine well without significant side effects. Unfortunately, over the past year, the patient was diagnosed with metastatic ovarian adenocarcinoma along with peritoneal carcinomatosis. She has been undergoing chemotherapy and radiation therapy.

In 2024, after experiencing persistent nausea during chemotherapy sessions, her oncologist initiated treatment with domperidone 10 mg three times daily. At this point, the patient had been on desvenlafaxine for 4 years, with the most recent dose increasing in September 2023. Within 1 week of commencing domperidone, the patient reported experiencing “brain zaps,” described as electrical jolts in her brain, and feelings of anxiety. She had previously experienced these sensations, typically when a desvenlafaxine dose was delayed or missed. However, in the past, these episodes were brief, intermittent, and infrequent, resolving quickly upon desvenlafaxine administration. Upon initiating domperidone, the brain zaps occurred within 30 min of dosing and persisted throughout the day. Upon discontinuation of domperidone, the brain zaps resolved almost immediately within a couple of days.

The patient reported that these brain zaps were similar to those experienced during venlafaxine dose reduction 5 years ago. As the brain zaps had resolved by the time of the follow-up clinical appointment, no further investigations were ordered. Clinical assessment revealed no changes in mood state or anxiety level leading up to or during the reported brain zaps. There was no evidence of psychotic symptoms. Apart from nausea and fatigue, the patient denied any other concerns during the episode of brain zaps. The patient reported no history of seizures or altered level of consciousness during the episode. The patient confirmed that the brain zaps did not recur following domperidone discontinuation.

## Discussion

This case highlights a potential drug–drug interaction between desvenlafaxine, an SNRI, and domperidone, a dopamine D2 receptor antagonist commonly used to manage nausea and vomiting. The patient, who had been stable on desvenlafaxine for many years, developed severe discontinuation-like symptoms (brain zaps, headaches, and anxiety) shortly after initiating domperidone despite adherence to the desvenlafaxine regimen. These symptoms resolved dramatically upon discontinuation of domperidone, suggesting a possible interaction between the two medications.

To explore the potential interaction between desvenlafaxine and domperidone, a comprehensive literature review was conducted using major academic databases, including PubMed, Google Scholar, and CINAHL. The search strategy employed relevant MeSH terms and keywords, such as “desvenlafaxine,” “domperidone,” “SNRI,” “; interaction,” “discontinuation syndrome,” and “dopamine-serotonin interaction.” Our literature review was expanded to include “withdrawal symptoms” and “antidepressant withdrawal” alongside “discontinuation syndrome,” yet no prior reports of a domperidone-desvenlafaxine interaction were identified, underscoring the uniqueness of this observation.

Despite this thorough search, no prior studies or case reports have specifically investigated the relationship between desvenlafaxine and domperidone or documented a similar interaction. The absence of published evidence highlights the novelty of the present case and underscores the need for further research to elucidate the mechanisms and clinical implications of this potential interaction.

A significant antidepressant discontinuation symptom is a form of paraesthesia referred to as “brain zaps,” which are uncomfortable and can feel like electric shocks. These sensations are likely linked to adrenergic withdrawal. As a result, SNRIs are more likely to cause brain zaps compared to SSRIs. Notably, venlafaxine has a disproportionately higher incidence of brain zaps, but paroxetine also shows similar reports, along with many other antidepressants.^22^

The patient’s presentation of anxiety and brain zaps following the initiation of domperidone, despite no changes in desvenlafaxine dosage or administration, raises the possibility of a drug interaction between domperidone and desvenlafaxine. While domperidone is primarily a peripheral dopamine receptor antagonist, its potential influence on central neurotransmitter systems cannot be entirely ruled out. Several mechanisms may explain this phenomenon:

Gastrointestinal effects on drug absorption: domperidone’s prokinetic effects on the gastrointestinal tract could alter the absorption kinetics of other drugs.^23^ By increasing gastric motility, it may speed up the emptying of the stomach, which can affect how quickly or how much of an orally administered drug is absorbed into the bloodstream. Depending on the drug, this can lead to either lower or higher blood concentrations. For example, if a drug requires slower gastric emptying for optimal absorption, such as desvenlafaxine, domperidone might reduce the absorption of that drug, potentially lowering its effectiveness.^24,25^ Desvenlafaxine is an extended-release tablet that is absorbed from the small intestine. Since domperidone will increase gastric motility, it will increase the rate at which desvenlafaxine passes through the small intestine, overall reducing the systemic absorption of desvenlafaxine. This is because the amount of time desvenlafaxine interacts with the absorption site in the small intestine is decreased. While the patient’s desvenlafaxine dose remained unchanged, changes in absorption could lead to subtherapeutic levels of the drug, mimicking discontinuation syndrome. This mechanism has been proposed for other prokinetic agents, such as metoclopramide, which have been associated with changes in the bioavailability of coadministered medications.^26^ Another critical point is that desvenlafaxine is primarily metabolized via conjugation (e.g. glucuronidation) with only minor involvement of CYP3A4 oxidation, and it exhibits minimal interaction with CYP450 enzymes as either a substrate or inhibitor.^27^ Domperidone, conversely, is metabolized predominantly by CYP3A4 and acts as a weak inhibitor of this enzyme.^28^ Given that desvenlafaxine’s metabolism is largely independent of CYP3A4, significant pharmacokinetic interactions via CYP450 pathways between these two medications are unlikely. Accordingly, no significant CYP450-mediated interactions are expected between domperidone and desvenlafaxine.

The withdrawal symptoms observed in this patient are consistent with the discontinuation syndrome of SNRIs, which can include brain zaps, insomnia, and hyperarousal. All three symptoms were observed in this patient. However, we recognize that alternative explanations, such as the effects of chemotherapy, radiation, or undetected CNS involvement, could confound the attribution of symptoms to a domperidone-desvenlafaxine interaction. Chemotherapy-induced nausea and neurotoxicity may mimic or exacerbate discontinuation-like symptoms. However, the temporal association with domperidone initiation, in the absence of prior similar complaints during desvenlafaxine therapy, supports our hypothesis as a contributing factor, though not definitively proven.

This case underscores the necessity for healthcare providers to consider potential pharmacodynamic drug interactions in addition to pharmacokinetic drug interactions. The majority of online databases adequately address pharmacokinetic drug interactions; however, there is a paucity of data regarding pharmacodynamic interactions.

The optimal approach to verifying this interaction would have been to measure plasma levels of desvenlafaxine before and after domperidone administration through blood sample analysis. However, this diagnostic test is not readily accessible to patients.

To our knowledge, this is the first reported case of domperidone potentially inducing discontinuation syndrome-like symptoms in a patient on stable SNRI therapy. This finding is significant because domperidone is widely used and generally considered to have minimal CNS effects. Clinicians should be aware of this potential interaction, particularly in patients with a history of antidepressant use or sensitivity to changes in neurotransmitter balance.

## Conclusion

This case report highlights a unique and previously unreported interaction between domperidone and desvenlafaxine, resulting in discontinuation syndrome in a patient on ongoing desvenlafaxine treatment. The temporal relationship between domperidone initiation, symptom onset, and symptom resolution upon discontinuation suggests a potential pharmacological interaction. While the exact mechanism remains unclear, possible explanations include dopamine-serotonin interactions and changes in drug absorption or CYP450 enzyme interactions. This case underscores the importance of considering drug interactions involving dopamine antagonists and SNRIs, even in the absence of direct pharmacological overlap. Further research is needed to explore the mechanisms underlying this interaction and to determine its clinical significance.
